# *SCN9A* variant in a family of mixed breed dogs with congenital insensitivity to pain

**DOI:** 10.1111/jvim.16610

**Published:** 2023-01-11

**Authors:** Rodrigo Gutierrez‐Quintana, Matthias Christen, Kiterie M. E. Faller, Julien Guevar, Vidhya Jagannathan, Tosso Leeb

**Affiliations:** ^1^ Small Animal Hospital, School of Biodiversity, One Health and Veterinary Medicine University of Glasgow Glasgow UK; ^2^ Institute of Genetics, Vetsuisse Faculty University of Bern Bern Switzerland; ^3^ Royal (Dick) School of Veterinary Studies The University of Edinburgh Midlothian UK; ^4^ Department of Clinical Veterinary Sciences, Vetsuisse Faculty University of Bern Bern Switzerland

**Keywords:** animal model, *Canis lupus familiaris*, genetics, neurology, precision medicine, sodium channel

## Abstract

**Background:**

Congenital insensitivity to pain (CIP) and hereditary sensory and autonomic neuropathies (HSANs) are a rare group of genetic disorders causing inability to feel pain. Three different associated variants have been identified in dogs: 1 in Border Collies, 1 in mixed breed dogs, and 1 in Spaniels and Pointers.

**Objectives:**

To clinically and genetically characterize CIP in a family of mixed breed dogs.

**Animals:**

Two mixed breed dogs from the same litter were independently presented: 1 for evaluation of painless fractures, and the other for chronic thermal skin injuries.

**Methods:**

Physical, neurological, and histopathological evaluations were performed. Whole genome sequencing of 1 affected dog was used to identify homozygous protein‐changing variants that were not present in 926 control genomes from diverse dog breeds.

**Results:**

Physical and neurological examinations showed the absence of superficial and deep pain perception in the entire body. Histopathological evaluations of the brain, spinal cord and sensory ganglia were normal. Whole genome sequencing identified a homozygous missense variant in *SCN9A*, XM_038584713.1:c.2761C>T or XP_038440641.1:(p.Arg921Cys). Both affected dogs were homozygous for the mutant allele, which was not detected in 926 dogs of different breeds.

**Conclusions and Clinical Importance:**

We confirmed the diagnosis of CIP in a family of mixed breed dogs and identified a likely pathogenic variant in the *SCN9A* gene. The clinical signs observed in these dogs mimic those reported in humans with pathogenic *SCN9A* variants causing CIP. This report is the first of a spontaneous pathogenic *SCN9A* variant in domestic animals.

**Abbreviations**
CIP: congenital insensitivity to pain
GATK: genome analysis toolkit
GDNF: glial cell‐derived neurotrophic factor
gVCF: genomic variant call format
HSAN: hereditary sensory and autonomic neuropathy
mRNA: messenger ribonucleic acid
NCBI: National Center for Biotechnology Information
OMIA: Online Mendelian Inheritance in Animals
OMIM: Online Mendelian Inheritance in Man
PCR: polymerase chain reaction
RETREG1: reticulophagy regulatory 1
SCN9A: sodium voltage‐gated channel alpha subunit 9
WGS: whole genome sequencing

## 1 INTRODUCTION

Pain is a sensory modality used to detect potential and real tissue damage, providing a survival advantage.^1^, ^2^ Genetic pain loss disorders are classified as congenital insensitivity to pain (CIP) or hereditary sensory and autonomic neuropathy (HSAN).^2^, ^3^ Congenital insensitivity to pain usually is defined by its congenital onset, whereas HSAN tends to develop gradually over time, but occasionally the difference is not clearly specified, and the terms can overlap.^2^ In all cases of CIP or HSAN, the consistent feature is decreased pain perception and resulting injuries.^2^, ^3^ In humans, seven forms of CIP and eight forms of HSAN have been described based on phenotype, and genetic variants in at least 26 genes have been reported.^2^, ^3^

Congenital insensitivity to pain and HSANs have been described previously in some dog breeds including French Spaniel,^4^, ^5^ English Springer Spaniel,^4^, ^5^ Pointer,^4^, ^6^, ^7^, ^8^ Border Collie,^9^, ^10^, ^11^, ^12^ Border Collie cross,^13^ Miniature Pincher,^14^ Long‐haired Dachshund,^15^, ^16^ Jack Russell Terrier,^17^ Fox Terrier,^18^ and a family of mixed breed dogs,^19^ but only three causal genetic variants have been identified to date. The first is an inversion disrupting *RETREG1* (*reticulophagy regulator 1*) in Border Collies and Border Collie crosses with HSAN (OMIA 002032‐9615).^9^, ^13^ The second is a missense variant in the same gene in a family of mixed breed dogs with HSAN (OMIA 002032‐9615).^19^ The third variant is a regulatory single base substitution in a lincRNA upstream of the *GDNF* (*glial cell‐derived neurotrophic factor*) gene encoding glial cell‐derived neurotrophic factor in Pointers, English Springer Spaniels and French Spaniels with acral mutilation syndrome (OMIA 001514‐9615).^4^

We investigated 2 mixed breed puppies from the same litter. One had tibial and fibular fractures and was weight‐bearing with the leg bending and no signs of pain. The other had chronic skin injuries caused by burns from sleeping in contact to the heating radiator. Here we describe the clinical presentation, histopathological features, outcome, and genetic investigations of these cases, in which we found a homozygous missense variant in *SCN9A*, XM_038584713.1:c.2761C>T or XP_038440641.1:(p.Arg921Cys). The *SCN9A* (*sodium voltage‐gated channel alpha subunit 9*) gene encodes the alpha subunit of the NaV1.7 voltage‐gated sodium channel, which is preferentially expressed in sensory neurons and plays a critical role in the generation and conduction of action potentials.^2^ Loss‐of‐function mutations in this gene have been associated with complete insensitivity to pain in humans.^2^

## 2 MATERIALS AND METHODS

### 2.1 Animals

Two related female mixed breed dogs were evaluated separately, the first 1 at 2 months of age (Case 1) and the second at 8 months of age (Case 2). They were from the same litter of reportedly healthy parents and some of the littermates also were reported to be healthy. Residual blood samples were retained from Case 2, and buccal swabs were collected from Case 1 for genetic investigations. Samples from the dam, sire or other littermates could not be obtained.

### 2.2 Necropsy examination

Owner consent was given for euthanasia and complete necropsy in Case 2. In addition to routine samples taken during necropsy (including brain and cervical, thoracic and lumbar spinal cord), representative samples from dorsal root ganglia (cervical, thoracic, and lumbar) were collected and fixed in 10% buffered formalin. Slices of formalin‐fixed samples were embedded in paraffin before staining with hematoxylin and eosin.

### 2.3 Sequencing and genotyping

#### 2.3.1 DNA extraction

Genomic DNA was isolated from EDTA blood and buccal swabs with the Maxwell RSC Whole Blood Kit and the RSC Buccal Swab DNA Kit, respectively, using a Maxwell RSC instrument (Promega, Dübendorf, Switzerland).

#### 2.3.2 Whole‐genome sequencing

An Illumina TruSeq PCR‐free DNA library with approximately 403 bp insert size from Case 2 was prepared. We collected 200 million 2 × 150 bp paired‐end reads on a NovaSeq 6000 instrument (20.8 × coverage). Mapping and alignment to the UU_Cfam_GSD_1.0 genome reference assembly were performed as described.^20^ The sequence data were deposited under the study accession PRJEB16012 and sample accession SAMEA110175953 at the European Nucleotide Archive.

#### 2.3.3 Variant calling

Variant calling was performed using GATK HaplotypeCaller^21^ in gVCF mode as described.^20^ To predict the functional effects of the called variants, SnpEff software^22^ together with NCBI annotation release 106 for the UU_Cfam_GSD_1.0 genome reference assembly was used. For variant filtering, we used 926 genetically diverse control dog genomes of different breeds (Table S1).

#### 2.3.4 Gene analysis

We used the UU_Cfam_GSD_1.0 dog reference genome assembly and NCBI annotation release 106. Numbering within the canine *SCN9A* gene corresponds to the NCBI RefSeq accession numbers XM_038584713.1 (mRNA) and XP_038440641.1 (protein).

#### 2.3.5 PCR and Sanger sequencing

The candidate variant *SCN9A*:c.2761C>T was genotyped by direct Sanger sequencing of PCR amplicons. A 325 bp PCR product was amplified from genomic DNA using AmpliTaqGold360Mastermix (Thermo Fisher Scientific, Waltham, MA, USA) and the primers 5′‐GAG TAA AGG CCA GTT CTT TGG A‐3′ (Primer F) and 5′‐CCT GGT AAC CCA GAA ACA TCA‐3′ (Primer R). Sanger sequences were analyzed using the Sequencher 5.1 software (GeneCodes, Ann Arbor, MI, USA).

#### 2.3.6 In silico functional predictions

The protein amino acid change caused by the candidate variant was assessed using multiple in silico prediction tools: PredictSNP, MutPred2 and SNPs & Go.^22^, ^23^, ^24^

## 3 RESULTS

### 3.1 Clinical description

Two related mixed breed puppies from the same litter were presented over a 6‐month period to the Small Animal Hospital of the University of Glasgow.

**FIGURE 1:**
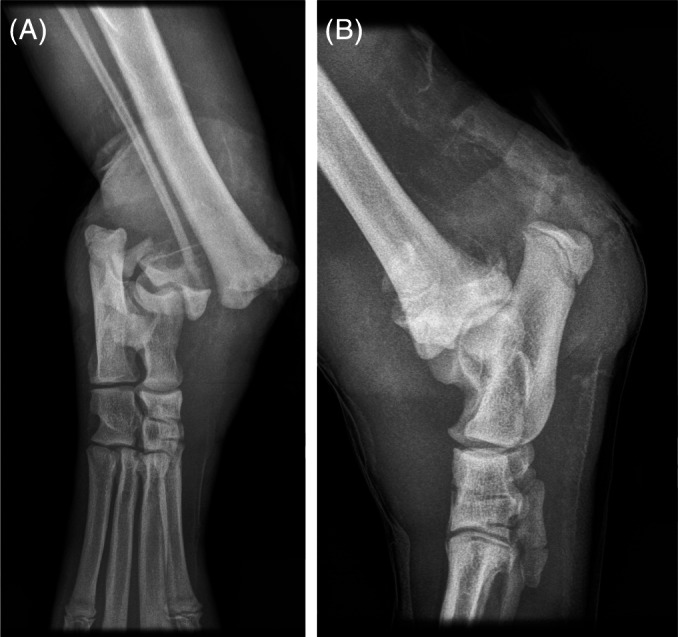
Cranio‐caudal (A) and lateral (B) radiograph of the right pelvic limb of case 1 showing the tibial and fibular fractures and soft tissue swelling

Case 1: A 2‐month‐old intact female puppy was presented with a 4‐day history of unusual bending of the right pelvic limb. The owner also mentioned the presence of small, round, superficial ulcers on the digital pads of both pelvic limbs for the last 4 weeks. Examination disclosed small superficial skin ulcers on digital pads IV and V. There was fluctuant swelling of the medial aspect of the right hock with associated instability, but no evidence of pain on palpation and manipulation. Neurological examination showed normal mentation and cranial nerve function. Gait assessment showed weight‐bearing lameness with lateral bending of the distal tibia. Postural reactions and segmental spinal reflexes were normal. Superficial and deep pain perception was absent over the entire body, with no evident response despite firm pressure with the forceps. Findings were consistent with a peripheral sensory neuropathy or CIP. Radiographs of the right tibia identified a displaced Salter‐Harris fracture type I of the distal tibia and a fibular fracture (Figure 1). Aspiration of the fluctuant swelling yielded purulent material with many neutrophils on microscopic examination. A diagnosis of infected tibial and fibular fractures was made, and the owner elected euthanasia.

Case 2: An 8‐month‐old intact female dog was presented with a history of chronic skin lesions. The owner reported that since adoption, at 6 weeks of age, the dog already had multiple scars in its skin. During the last 8 weeks, the dog developed large and deep skin lesions in the dorsolateral thoracic region bilaterally. Initial dermatological investigations did not identify a cause for the lesions. Since then, the owner noticed that these lesions were caused by getting burned while sleeping in contact with a heating radiator. Physical examination was unremarkable, except for multiple scars in the skin and the large erosion in the dorsolateral thoracic region. Multiple blood pressure measurements were normal. Neurological examination identified normal mentation, cranial nerve function and gait. Postural reactions and segmental spinal reflexes were normal. Superficial and deep pain perception was absent over the entire body, with no evident response despite firm pressure with the forceps. Findings were consistent with a peripheral sensory neuropathy or CIP. Results of a CBC and serum biochemistry profile were normal. The dog received open wound management (i.e., lavages, debridement, PO antibiotics [cephalexin], an anti‐inflammatory drug [meloxicam], and bandaging) and the owners were instructed to avoid any activities that could cause injury and to protect the dog from contact with the heating radiators. Despite these precautions, the dog developed multiple severe skin injuries over the next 2 months, and the owners elected euthanasia.

### 3.2 Histopathology

No macroscopic or microscopic abnormalities were detected in any of the tissues examined, except for the skin lesions previously reported.

### 3.3 Sequencing, genotyping and protein expression

Because the clinical and neurological findings of these cases resembled CIP previously described in humans, and the parents were reported to be clinically unaffected, we hypothesized that the phenotype in the affected dogs was caused by monogenic autosomal recessive mode of inheritance. We sequenced the genome of Case 2 and searched for private homozygous variants that were not present in the genome sequences of 926 control dogs of diverse breeds (Table 1 and Tables S1 and S2).

**TABLE 1:** Homozygous variants in case 2, filtered against 926 control genomes

Filtering step	Variants
All variants in the affected dog	3 036 781
Private variants	589
Protein‐changing private variants	8

**FIGURE 2:**
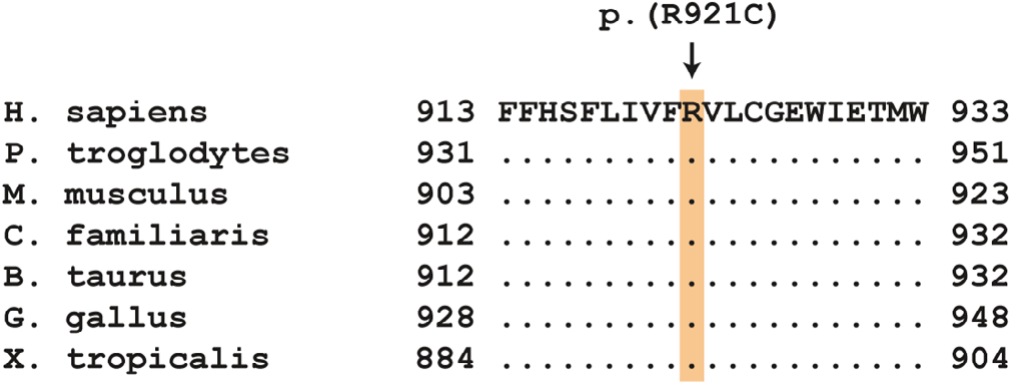
Multiple‐species alignment of the *SCN9A* amino acid sequences of the second intramembrane pore forming domain harboring the p.(R921C) variant. The variant affects a highly conserved arginine residue. Accession numbers: human (*Homo sapiens*) NP_001352465.1; chimpanzee (*Pan troglodytes*) XP_003309333.2; mouse (*Mus musculus*) NP_061340.2; dog (*Canis familiaris*) XP_038440641.1; domestic cattle (*Bos Taurus*) NP_001104257.1; chicken (*Gallus gallus*) XP_004942840.1; frog (*Xenopus tropicalis*) XP_002939316.2

The resulting variants were prioritized according to functional knowledge of the affected genes. The bioinformatics analysis identified a single homozygous private protein‐changing variant in a functional candidate gene. The variant was located in the *SCN9A* (*sodium voltage‐gated channel alpha subunit 9*) gene. It can be designated chr36:11652662G>A or XM_038584713.1:c.2761C>T and is predicted to result in an amino acid substitution in a highly conserved region of the encoded alpha subunit of the NaV1.7 sodium channel, XP_038440641.1:(p.Arg921Cys) (Figure 2).

The arginine‐to‐cysteine substitution was predicted to be deleterious by all used prediction algorithms (PredictSNP probability for pathogenicity 87%, MutPred2 score: 0.923, SNP&GO disease probability: 76%). Furthermore, MutPred2 predictions included “altered ordered interface” and “altered transmembrane protein” with probabilities of 0.28 and 0.25, and with *P*‐values of 5.9 × 10^−3^ and 1.4 × 10^−3^, respectively.

The other seven private protein‐changing variants were not located in genes known to cause similar phenotypes in humans, mice, or domestic animals.

**FIGURE 3:**
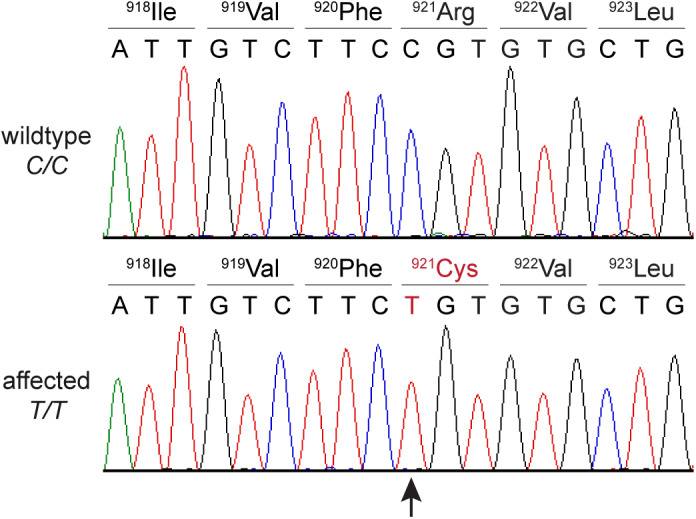
Details of the SCN9A:c.2761C>T variant (p.Arg921Cys). Representative electropherograms of a wild type dog and an affected dog are shown. The amino acid translations of the wild type and mutant alleles are indicated

We confirmed the presence of the *SCN9A* variant in a homozygous state in Cases 1 and 2 by Sanger sequencing (Figure 3).

## 4 DISCUSSION

We describe two littermate mixed breed dogs with the inability to react to noxious stimuli and secondary injuries, including fractures and burns. We identified a likely candidate disease‐causing variant, *SCN9A*:p.(Arg921Cys). Nociception refers to neural encoding of impending or actual tissue damage and pain refers to the subjective experience of actual or impending harm.^1^

The *SCN9A* (*sodium voltage‐gated channel alpha subunit 9*) gene encodes the alpha subunit of the NaV1.7 sodium channel. Gain of function variants produce impaired inactivation or enhanced resurgent current of the sodium channel and induce increased excitability in the dorsal root ganglia neurons, resulting in neuropathic pain and variable clinical phenotypes in humans such as primary erythromelalgia (OMIM# 133020), paroxysmal extreme pain disorder (OMIM# 167400) and hereditable small fiber neuropathy (OMIM# 133020). The *SCN9A* loss of function results in inability to feel pain and the human clinical phenotypes are congenital insensitivity to pain (OMIM# 243000) and hereditary sensory autonomic neuropathy type IID (OMIM# 243000).^2^, ^3^, ^25^, ^26^, ^27^ A recent study indicated that nociceptor activity at the level of the dorsal root ganglia is largely unaffected by NaV1.7 and suggested a critical locus of analgesia in the central terminal and not in the periphery as thought previously.^28^ The *SCN9A* gene also plays a role in seizures and epilepsy with some variants linked to Dravet Syndrome (OMIM# 607208) and febrile seizures.^29^

The clinical phenotype in humans with CIP caused by variants in *SCN9A* is characterized by anosmia and injuries associated with complete lack of pain sensation. The dogs of our study suffered from painless fractures and burns, which are hallmarks of the phenotype in humans. Anosmia is difficult to identify clinically in dogs, especially if present since birth, and we are not sure if it was present in these cases. When compared with previous reports of HSAN and CIP in dogs, the dogs of our study share more similarities with the Miniature Pinscher,^14^ Pointer,^4^, ^6^, ^7^, ^8^ Spaniel,^4^, ^5^ and Fox Terrier,^18^ that presented with loss of pain sensation, but no proprioceptive deficits or autonomic signs. In contrast, the HSAN reported in the long‐haired Dachshund,^15^, ^16^ Jack Russell Terrier,^17^ Border Collie,^9^, ^10^, ^11^, ^12^, ^13^ and a family of mixed breed dogs^19^ was associated with other neurological deficits including proprioceptive deficits and signs of autonomic dysfunction. An important difference in the dogs of our report is that automutilation was not a feature, but severe injuries were caused by external sources. Finally, the young age of presentation in our patients is consistent with CIP.

Several variants in *SCN9A* causing CIP have been identified in humans. Congenital insensitivity to pain causing loss of function variants mostly consist of nonsense, splice site, and frameshift variants, but also include some missense variants.^26^ The missense variant identified in the affected dogs of our report changes a highly conserved arginine residue in 1 of the 4 intramembrane pore‐forming domains of the NaV1.7 alpha subunit.^30^ The change from the positively‐charged arginine residue to an uncharged cysteine residue with a reactive thiol group in this region might disrupt sodium channel functionality by altering sodium ion selectivity and conductivity, as was suggested previously.^31^

The XP_038440641.1:(p.Arg921Cys) was predicted to be deleterious by all used in silico prediction tools. An identical amino acid exchange in the corresponding human protein, NP_001352465.1:p.Arg922Cys, has been observed in compound heterozygosity with a known disease‐causing variant in a patient with HSAN.^32^ The discovery of a homologous change in the dog now provides additional evidence for the pathogenicity of these variants in both species. Finally, the absence of the *SCN9A*:c.2761C>T variant in >900 control genomes and homozygosity in both cases provides additional support and make this variant a compelling candidate disease‐causing variant in the 2 affected puppies.

Currently, no cure exists for this condition, and treatment is supportive by early detection, prevention and management of any injuries sustained.^33^ Both of the dogs in our study presented with multiple and severe injuries, and owners elected euthanasia at a young age. In humans, many people with CIP do not survive childhood because of recurrent injuries, such as self‐injury, burns, repeated fractures, osteomyelitis, and accidental death.^34^

Our study had some limitations. First, because of the lack of pedigree data and inability to obtain samples from relatives, we were not able to confirm the mode of inheritance. Nevertheless, the severity of the phenotype and the reportedly healthy parents make monogenic autosomal recessive the most likely mode of inheritance. Second, no histopathology of the peripheral nerves or autonomic ganglia was performed, and doing so could have helped characterize a form of HSAN or CIP. Finally, no nerve conduction or electromyographic studies were performed.

To our knowledge, our study is the first description of CIP associated with a *SCN9A* variant in domestic animals. The clinical features of this disease closely resemble the human phenotype, suggesting the *SCN9A*:p.Arg921Cys variant as a compelling candidate disease‐causing variant. It is not currently known whether the causal variant arose in the recent ancestry of the affected dogs described in our study or whether it arose more distantly, in which case it might be segregating in the wider canine population.

## CONFLICT OF INTEREST

Authors declare no conflict of interest.

## OFF‐LABEL ANTIMICROBIAL DECLARATION

Authors declare no off‐label use of antimicrobials.

## INSTITUTIONAL ANIMAL CARE AND USE COMMITTEE (IACUC) OR OTHER APPROVAL DECLARATION

Approved by the local ethical committee of the University of Glasgow, School of Veterinary Medicine.

## HUMAN ETHICS APPROVAL DECLARATION

Authors declare human ethics approval was not needed for this study.

## Supporting information


**Table S1.** Public genomes used for variant filtering.

**Table S2.** Private homozygous variants compared to 926 control genomes of different dog breeds.
